# MEASUREMENT EQUIVALENCE OF THE POSITIVE AND NEGATIVE AFFECT SCHEDULE BETWEEN YOUNG-MIDDLE-AGED AND OLDER ADULTS

**DOI:** 10.1093/geroni/igz038.2591

**Published:** 2019-11-08

**Authors:** Jeongwoo Lee, En-Jung Shon

**Affiliations:** 1 Case Western Reserve University, Cleveland, Ohio, United States; 2 Miami University, Oxford, Ohio, Oxford, Ohio, United States

## Abstract

A Short form of the Positive and Negative Affect Schedule (PANAS-SF) has been widely used to measure of affect in diverse cultural groups. Limited studies have been evaluated the measurement equivalence test of PANAS-SF in diverse age groups. This study examined whether parameters in the measurement model (two-factor model: positive and negative affect) is equivalent across the two age generations (young-middle aged: <65 years [n=1,122]; older adults : ≥65 years [n=1,817]). The sample was obtained from the 2012 Health and Retirement Study and Multiple Group Analysis was performed. The five items of determined, enthusiastic, inspired, alert, and excited reflected positive affect; and the five items of afraid, upset, scared, nervous, and distressed reflected negative affect. The configural model reported acceptable fit (X2= 904.98 [df = 64, p < .001], X 2/df =14.14, CFI =.93, GFI=.94, RMSEA=.06 [90% CI=.06 - .07]). When all factor loadings were constrained, it indicated measurement non-invariance status between young-middle aged and older adults (ΔX 2 = 56.03, Δdf = 8, p< .001, CFI=.93, ΔCFI=.004). Given findings of non-invariance on the full constrained model, the invariance test of each factor loading was performed additionally. Majority of negative items (Afraid, upset, scared, and nervous) and several positive items (determined and excited) were nonequivalent between the two groups. Variances in the measure between two age groups raise a number of issues for future research on affect assessment, suggesting cautious using of PANAS-SF in older adults.

